# Clinical Comparison of 4D Phaco and Torsional Ultrasound Phacoemulsification Platforms: Association with Cumulative Dissipated Energy and Postoperative Inflammatory Response

**DOI:** 10.3390/jcm15176843

**Published:** 2026-09-03

**Authors:** Hisaharu Suzuki

**Affiliations:** Zengyo Suzuki Eye Clinic, Fujisawa 251-0871, Kanagawa, Japan; s5054@nms.ac.jp

**Keywords:** phacoemulsification, cataract surgery, 4D Phaco, torsional ultrasound, cumulative dissipated energy, aqueous flare, laser flare photometry, corneal endothelial cell loss, hybrid phacoemulsification tip

## Abstract

**Background/Objectives:** Four-dimensional Phaco and torsional ultrasound represent distinct approaches to phacoemulsification, and both platforms are compatible with a hybrid polymer-coated tip designed to reduce posterior capsule contact risk. Clinical comparative data evaluating both ultrasound efficiency and postoperative inflammatory response remain limited. **Methods:** We retrospectively compared 149 eyes of 139 patients undergoing phacoemulsification with the UNITY (4D Phaco) or CENTURION (torsional ultrasound) platform, each combined with a standard metal or hybrid polymer-coated tip, evaluating cumulative dissipated energy (CDE), postoperative aqueous flare, endothelial cell loss, visual acuity, and surgical time. Because CENTURION and UNITY were used during non-overlapping periods, the device comparison reflects a historical rather than concurrent design; tip-use periods overlapped substantially for CENTURION but only briefly (approximately three days) for UNITY. A 2 × 2 factorial regression model with heteroscedasticity-consistent (HC3) robust standard errors estimated device and tip effects and their interaction. **Results:** UNITY was associated with 52.8% lower geometric mean CDE (GMR 0.472; 95% CI, 0.413–0.539; *p* < 0.0001) and 31.6% lower 1-month aqueous flare (GMR 0.684; 95% CI, 0.580–0.806; *p* < 0.0001) than CENTURION. The hybrid tip was associated with 35.9% higher CDE (GMR 1.359; 95% CI, 1.190–1.551; *p* < 0.0001) but no significant flare effect. Endothelial cell loss, day-1 flare, visual acuity, and surgical time did not differ significantly by device or tip, with no device × tip interaction for any outcome (all *p* ≥ 0.073). **Conclusions:** UNITY was associated with substantially lower CDE and lower postoperative aqueous flare than CENTURION, without a demonstrable difference in endothelial cell loss or visual outcome, and without significant modification by tip type. Because the device comparison was historical rather than concurrent, these findings should be regarded as hypothesis-generating pending confirmation with concurrent device allocation.

## 1. Introduction

Phacoemulsification remains the standard technique for cataract extraction, and reducing cumulative dissipated energy (CDE) while preserving surgical efficiency remains a central goal of ultrasound modality development [1]. Torsional ultrasound reduced surgeon-generated tip travel and procedure time relative to longitudinal ultrasound by improving nuclear followability [2], and 4D Phaco extends this principle through multidirectional tip movement that generates a repeatable volumetric cutting pattern. The CENTURION Vision System (CENTURION, Alcon Vision LLC, Fort Worth, TX, USA) uses OZil torsional ultrasound, whereas the UNITY Vitreoretinal Cataract System (UNITY, Alcon Vision LLC) uses 4D Phaco. Bench studies report that 4D Phaco reduces ultrasound time, CDE, and total delivered energy by approximately 40–50% relative to torsional ultrasound [1,3], and we have separately shown, using the slit side view technique in a porcine-eye occlusion-break model, that the UNITY platform maintains greater anterior chamber stability than CENTURION with the Active Sentry handpiece [4]. Whether these bench-level advantages translate into clinical outcomes, however, remains unknown, and clinical comparative data on 4D Phaco versus torsional ultrasound are limited.

Both platforms are also compatible with a hybrid polymer-coated tip developed to reduce posterior capsule contact risk. Bench testing indicates that the hybrid tip tolerates greater force before capsule rupture than a standard metal tip [5,6]. Furthermore, clinical experience suggests acceptable ultrasound parameters and safety with this tip [7,8]. The clinical energy and inflammatory profile of the hybrid tip relative to a standard metal tip, however, has not been characterized across different ultrasound platforms within the same cohort.

Corneal endothelial cell loss and postoperative aqueous flare are complementary but biologically distinct markers of surgical stress, and both are relevant to comparing platforms at the tissue level. Endothelial injury is multifactorial: nuclear firmness has been reported as a stronger independent risk factor than ultrasound energy itself [9], and cumulative dissipated energy correlates only imperfectly with cell loss across fragmentation techniques [10,11]. Aqueous flare, measured objectively by laser flare photometry, instead reflects blood–aqueous barrier disruption and intraocular inflammatory stress [12]; it is influenced more by preoperative flare and cataract density than by ultrasound energy itself [13], and has been used to help stratify the risk of complications such as cystoid macular edema [14,15].

Given these considerations, we performed a retrospective clinical comparison of the UNITY and CENTURION platforms, each with a standard metal tip and a hybrid polymer tip, examining CDE, postoperative aqueous flare, corneal endothelial cell loss, visual outcome, and surgical time. In addition to a four-group omnibus comparison, we used a 2 × 2 factorial (device × tip) model to separately estimate device and tip main effects and to test for a device × tip interaction, an analytic approach that, to our knowledge, has not previously been applied to this platform-and-tip combination.

## 2. Materials and Methods

### 2.1. Study Design

This retrospective, single-center observational study included eyes that underwent phacoemulsification and intraocular lens implantation at Zengyo Suzuki Eye Clinic during the study period. Eligible eyes were classified into four groups according to the phacoemulsification platform and tip used: UNITY with a standard metal tip (UNITY Standard), UNITY with a hybrid polymer-coated tip (UNITY Hybrid), CENTURION with a standard metal tip (CENTURION Standard), and CENTURION with a hybrid polymer-coated tip (CENTURION Hybrid). In addition to the four-group comparison, the groups were analyzed using a 2 × 2 factorial design comprising device type (UNITY vs. CENTURION) and tip type (standard metal vs. hybrid polymer-coated).

The CENTURION platform was used exclusively before the UNITY platform was introduced into routine clinical practice. Surgical dates confirmed that the CENTURION cohort (standard tip: 2 December 2024–23 January 2025; hybrid tip: 25 September 2024–23 January 2025) and the UNITY cohort (standard tip: 12 November–17 December 2025; hybrid tip: 15 December 2025–26 January 2026) were performed during essentially non-overlapping calendar periods, separated by approximately 9 months. This interval reflects a deliberate transition period during which the UNITY platform’s fluidic settings, including intraocular pressure calibration, were being finalized for routine clinical use; eyes operated on during this calibration period were not included in the study, so that all UNITY cases analyzed here reflect stable, finalized device settings. Within each device, standard-tip and hybrid-tip cases were performed with substantial temporal overlap for CENTURION; for UNITY, however, the standard-tip and hybrid-tip cohorts overlapped for only a brief period (approximately three days) near the transition to the hybrid tip, as detailed above. The UNITY tip comparison in particular should also be interpreted with this near-sequential enrollment in mind; this distinction is addressed further in the Discussion and Limitations.

Only information obtained during routine clinical care was used, and no additional examination or intervention was performed for this study. The study was conducted in accordance with the Declaration of Helsinki and was approved by the Institutional Review Board of the Zengyo Suzuki Eye Clinic (IRB registration No. 21000029, Ministry of Health, Labour and Welfare Ethics Review Committee Reporting System; institutional protocol No. 12; approval date: 24 June 2026). Patients had provided general consent for the use of their clinical data for research purposes as part of routine clinical care; owing to the retrospective design of this specific study, the requirement for additional written informed consent was waived, and information regarding the study was disclosed through an opt-out procedure.

### 2.2. Patients

Medical records of consecutive patients who underwent routine cataract surgery with intraocular lens implantation at Zengyo Suzuki Eye Clinic between September 2024 and January 2026 were retrospectively reviewed.

Eyes were included if they had age-related cataract of Emery-Little grade 3 or lower and underwent uncomplicated phacoemulsification using either the UNITY or the CENTURION, with either a standard metal tip or a hybrid polymer-coated tip. The Emery-Little grade 3 cutoff was applied to limit variability in lens density across the four groups.

Eyes were excluded if they had previous intraocular surgery, corneal disease affecting endothelial cell measurements, glaucoma requiring previous filtering surgery, uveitis or other anterior-segment inflammatory disease, ocular trauma, pseudoexfoliation syndrome with significant zonular instability, shallow anterior chamber, poor pupillary dilation, intraoperative complications (including posterior capsule rupture or vitreous loss), combined surgical procedures, or incomplete clinical data at the 1-month postoperative visit.

**Figure 1:**
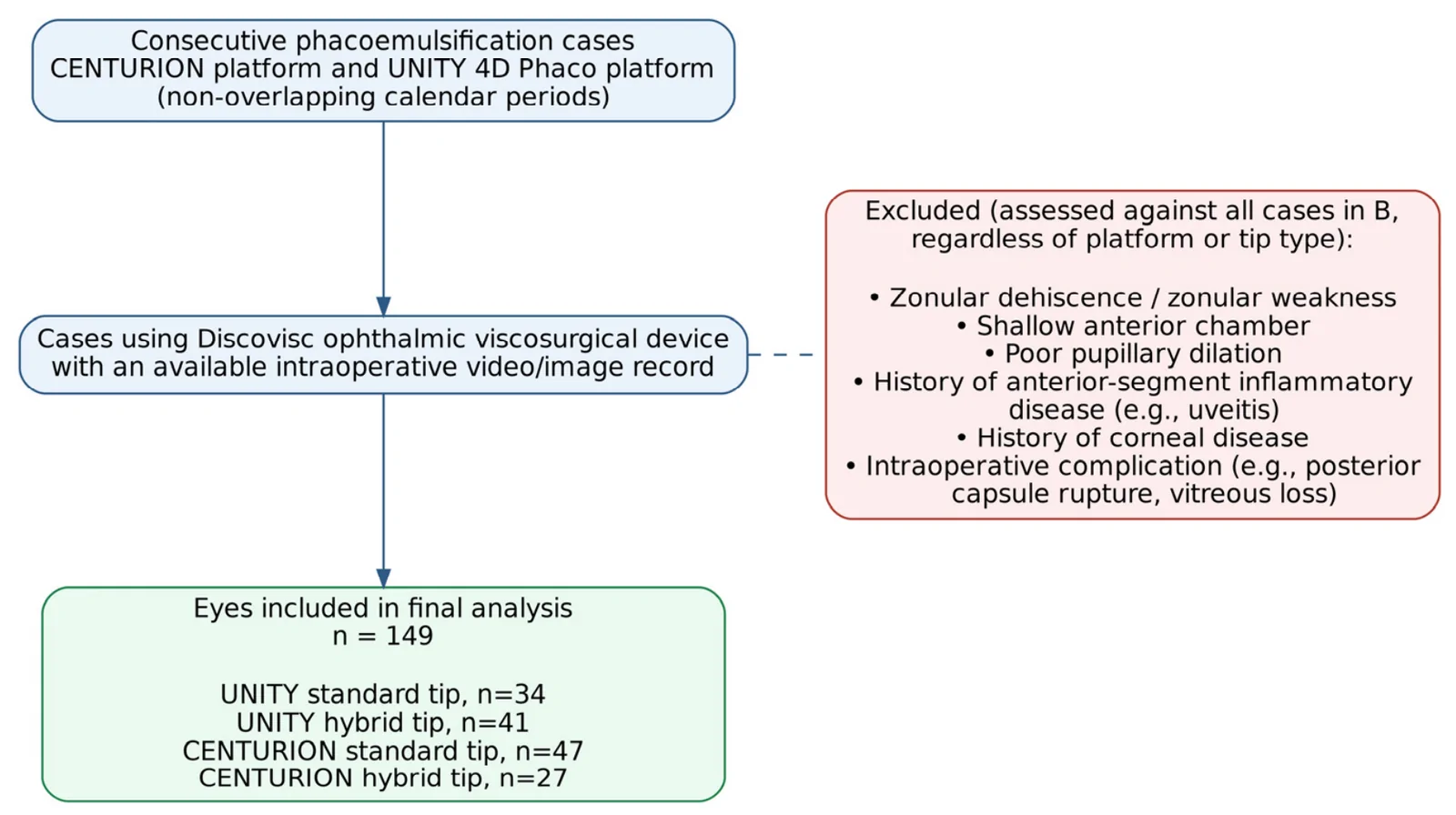
Participant flow diagram. All eyes were drawn from consecutive phacoemulsification cases performed with the CENTURION or UNITY 4D Phaco platform using the Discovisc ophthalmic viscosurgical device and having an available intraoperative video/image record; the exact number of cases meeting this screening step was not separately tabulated and is noted as a limitation. Eyes were then excluded per the criteria listed in Section 2.2 (previous intraocular surgery; corneal disease affecting endothelial cell measurements; glaucoma requiring previous filtering surgery; uveitis or other anterior-segment inflammatory disease; ocular trauma; pseudoexfoliation syndrome with significant zonular instability; shallow anterior chamber; poor pupillary dilation; intraoperative complications including posterior capsule rupture or vitreous loss; combined procedures; or incomplete 1-month data), applied identically regardless of platform or tip type. The resulting 149 eyes constitute the analytic cohort reported in Tables 2–4.

When both eyes of a patient met the eligibility criteria, both eyes were included in the analysis. Across the full cohort of 149 eyes, 139 unique patients were represented. Twenty of the 149 eyes (from 10 patients) were paired within patients: 2 patients contributed both eyes to the UNITY Standard group, 7 patients contributed both eyes to the UNITY Hybrid group, and 1 patient contributed one eye to the UNITY Standard group and the fellow eye to the UNITY Hybrid group after the hybrid tip was introduced for that platform. No patient contributed both eyes to the CENTURION Standard or CENTURION Hybrid groups. The primary statistical analyses (Section 2.5) treated eyes as independent units of observation; the resulting modest violation of the independence assumption for these within-patient eye pairs, which were concentrated in the UNITY Standard and UNITY Hybrid groups, is addressed in the Limitations. The participant selection process is summarized in Figure 1.

### 2.3. Surgical Procedure

All surgeries were performed by a single experienced surgeon (H.S.) using a standardized phacoemulsification technique through a 2.4-mm transconjunctival single-plane corneoscleral incision under topical anesthesia.

Phacoemulsification was performed using either the UNITY equipped with 4D Phaco technology or the CENTURION with torsional ultrasound, each combined with a standard metal tip or a hybrid polymer-coated tip, resulting in four study groups: UNITY Standard, UNITY Hybrid, CENTURION Standard, and CENTURION Hybrid. All procedures were performed using an Active Sentry intraocular pressure-sensing handpiece on both platforms.

The choice of surgical platform reflected the chronological introduction of the UNITY system into routine clinical practice (Section 2.1), whereas the selection of the phaco tip, once the hybrid tip became available for a given platform, followed the surgeon’s concurrent standard clinical practice for that platform. Apart from the phacoemulsification platform and tip, the surgical technique and perioperative management were consistent throughout the study period.

A continuous curvilinear capsulorhexis was performed in all eyes, followed by hydrodissection and hydrodelineation. Nuclear fragmentation was performed using a stop-and-chop technique: a central groove was sculpted to approximately mid-nuclear depth, the phaco tip was then embedded at the base of the groove, and the nucleus was divided into two hemi-nuclei by a chop maneuver. Each hemi-nucleus was further divided into two fragments by vertical chop, yielding three to four nuclear fragments, which were then removed by phacoemulsification and aspiration. Discovisc (Alcon Vision LLC) was used as the ophthalmic viscosurgical device during phacoemulsification, and a cohesive OVD was used to facilitate intraocular lens insertion after nucleus and cortex removal. A foldable single-piece intraocular lens, sourced from several manufacturers, was implanted in the capsular bag in all eyes; lens selection (including monofocal, enhanced monofocal, extended depth-of-focus, and trifocal designs) followed the surgeon’s routine clinical practice and patient preference and was not standardized across the cohort.

For the CENTURION platform, torsional ultrasound power was set to 40%, with vacuum increasing from 80 to 310 mmHg and aspiration flow rate increasing from 21 to 22 mL/min between the grooving/sculpting phase and the nucleus removal phase; intraocular pressure was maintained at 20 mmHg. For the UNITY platform, 4D Phaco power was set to 40%, vacuum increased from 80 to 350 mmHg, aspiration flow rate was maintained at 22 mL/min (the same as the CENTURION platform), and intraocular pressure was maintained at 20 mmHg throughout the grooving and nucleus removal phases. Incision size and intraocular lens implantation technique were standardized according to the routine protocol of our institution.

All patients received an identical standardized postoperative topical regimen, irrespective of group: moxifloxacin, 0.1% betamethasone phosphate, and bromfenac from postoperative day 1 through 2 weeks, followed by 0.1% fluorometholone and bromfenac from 2 weeks through 1 month. Because this regimen did not differ across the four groups, it does not account for the between-group differences in postoperative aqueous flare observed in this study.

### 2.4. Outcome Measures

The primary outcome measures were cumulative dissipated energy (CDE), postoperative aqueous flare, and corneal endothelial cell loss. CDE was the console-reported cumulative dissipated energy value automatically logged by each platform’s software (UNITY or CENTURION) at case completion; the underlying calculation uses platform-specific algorithms, and we did not independently verify formula-level equivalence between the two consoles’ CDE outputs. Total ultrasound time (as distinct from total surgical time) was not separately recorded in this retrospective dataset, so we were unable to report it as an additional, potentially more directly comparable energy-exposure metric; accordingly, CDE is interpreted here strictly as the console-reported metric for each platform and not as a directly validated measure of equivalent physical energy delivered across platforms. Secondary outcome measures were total surgical time and corrected distance visual acuity (CDVA). Preoperative anterior chamber depth (ACD), central corneal thickness (CCT), and axial length (AL) were recorded as baseline biometric characteristics used to assess comparability among the four groups; because they were not reassessed postoperatively, they were not treated as outcome measures.

Corneal endothelial cell density (ECD) was measured preoperatively and at 1 month postoperatively using a noncontact specular microscope (CEM-530, NIDEK Co., Ltd., Gamagori, Japan), and endothelial cell loss was calculated as the percentage reduction from the preoperative value.

Aqueous flare was measured at postoperative day 1 and month 1 using a laser flare meter (FM-600α, Kowa Co., Ltd., Tokyo, Japan) and expressed in photon counts per millisecond (pc/ms).

Preoperative ACD and CCT were measured using anterior segment optical coherence tomography (CASIA2 Advance, Tomey Corporation, Nagoya, Japan), and AL was measured using swept-source optical coherence tomography (ARGOS, Alcon Vision LLC).

Corrected distance visual acuity (CDVA) was assessed at 1 month postoperatively and converted to logarithm of the minimum angle of resolution (logMAR) units for statistical analysis.

Cumulative dissipated energy (CDE) and surgical time were automatically recorded by the phacoemulsification system at the completion of each procedure.

### 2.5. Statistical Analysis

Continuous variables are presented as mean ± standard deviation (SD), and categorical variables are presented as frequencies and percentages. Continuous variables were first assessed for normality within each of the four groups using the Shapiro–Wilk test, and for homogeneity of variance across groups using the Brown–Forsythe (median-based) modification of Levene’s test. When both assumptions were met, a one-way analysis of variance (ANOVA) was used for the omnibus four-group comparison, with Tukey’s honestly significant difference (HSD) test applied for pairwise post hoc comparisons. When either assumption was violated, the Kruskal–Wallis test was used for the omnibus comparison; when this was significant, pairwise Wilcoxon rank-sum tests with Bonferroni correction were performed as post hoc tests. Categorical variables (sex, operated eye) were compared among the four groups using the chi-square test.

To separately estimate the effect of device (UNITY vs. CENTURION) and tip (hybrid polymer vs. standard metal) and to test for a device × tip interaction, the four groups were additionally recoded into a 2 × 2 factorial design. Linear regression models with heteroscedasticity-consistent (HC3) robust standard errors were fitted for each outcome, with device, tip, and their interaction as predictors. Outcomes with right-skewed, heteroscedastic distributions (day-1 flare, 1-month flare, CDE, and total surgical time) were natural-log transformed before modeling; exponentiated coefficients are reported as geometric mean ratios (GMR) with 95% confidence intervals. When the device × tip interaction term was not statistically significant, the additive (main-effects-only) model was used to obtain the reported device and tip effect estimates. All statistical tests were two-sided, and *p* < 0.05 was considered statistically significant. Analyses were performed using R software (version 4.3.3; R Foundation for Statistical Computing, Vienna, Austria).

Eyes, rather than patients, were used as the unit of analysis, and the tests above assume independent observations. As described in Section 2.2, 20 of the 149 eyes (from 10 patients) were paired within patients, concentrated in the UNITY Standard and UNITY Hybrid groups; this clustering was not modeled explicitly in the primary factorial analyses. To assess the impact of this clustering, we performed two sensitivity analyses: (1) the factorial models were refitted with cluster-robust standard errors (CR1) using patient as the clustering unit, and (2) the factorial models were refitted after retaining only one randomly selected eye per patient (*n* = 139 eyes). Results of both sensitivity analyses are reported alongside the primary results in Section 3. As an additional sensitivity analysis for 1-month ECD, we fitted an ANCOVA model with baseline (preoperative) ECD as a covariate alongside device, tip, and their interaction, using HC3 robust standard errors, to complement the percentage-change analysis reported above.

### 2.6. Use of Artificial Intelligence

Generative artificial intelligence (AI) tools (Claude, Anthropic; ChatGPT, OpenAI) were used during manuscript preparation solely to assist with language editing, improvement of manuscript clarity and readability, and graphical presentation of figures. These tools were not used for study design, patient selection, data collection, statistical analysis, interpretation of the results, or scientific decision-making. All statistical analyses were designed, performed, and interpreted by the author, who takes full responsibility for their accuracy and for all scientific conclusions.

## 3. Results

A total of 149 eyes of 139 patients were included in the analysis: 34 in the UNITY Standard group, 41 in the UNITY Hybrid group, 47 in the CENTURION Standard group, and 27 in the CENTURION Hybrid group. Baseline characteristics, including age, sex distribution, operated eye, preoperative corneal endothelial cell density (ECD), preoperative anterior chamber depth (ACD), preoperative central corneal thickness (CCT), and axial length, are summarized in Table 1. Although no omnibus comparison reached statistical significance (all *p* > 0.05), these *p* values should not be interpreted as establishing baseline equivalence among the groups; numerical differences were present, including in axial length (*p* = 0.065).

**Table 1:** Baseline characteristics by device/technique group.

Characteristic	UNITY Standard (*n* = 34)	UNITY Hybrid (*n* = 41)	CENTURION Standard (*n* = 47)	CENTURION Hybrid (*n* = 27)	*p* Value
Age, y (mean ± SD)	72.7 ± 7.7	72.7 ± 6.5	73.5 ± 7.7	71.1 ± 8.7	0.626
Sex, F/M	25/9	29/12	30/17	20/7	0.736
Operated eye, L/R	17/17	19/22	25/22	15/12	0.877
Preoperative ECD, cells/mm^2^ (mean ± SD)	2726.8 ± 301.7	2834.1 ± 268.4	2761.6 ± 296.5	2742.3 ± 329.0	0.408
Preoperative ACD, mm (mean ± SD)	2.70 ± 0.38	2.78 ± 0.31	2.75 ± 0.44	2.74 ± 0.38	0.706
Preoperative CCT, µm (mean ± SD)	537.1 ± 31.2	533.1 ± 43.4	534.2 ± 32.9	531.0 ± 41.1	0.975
Axial length, mm (mean ± SD)	24.48 ± 1.41	25.00 ± 1.68	24.14 ± 1.74	24.39 ± 1.97	0.065

ECD = endothelial cell density; ACD = anterior chamber depth; CCT = central corneal thickness. Age and preoperative ECD were compared with one-way ANOVA; sex and eye laterality were compared with the chi-square test; all other variables were compared with the Kruskal–Wallis test.

**Figure 2:**
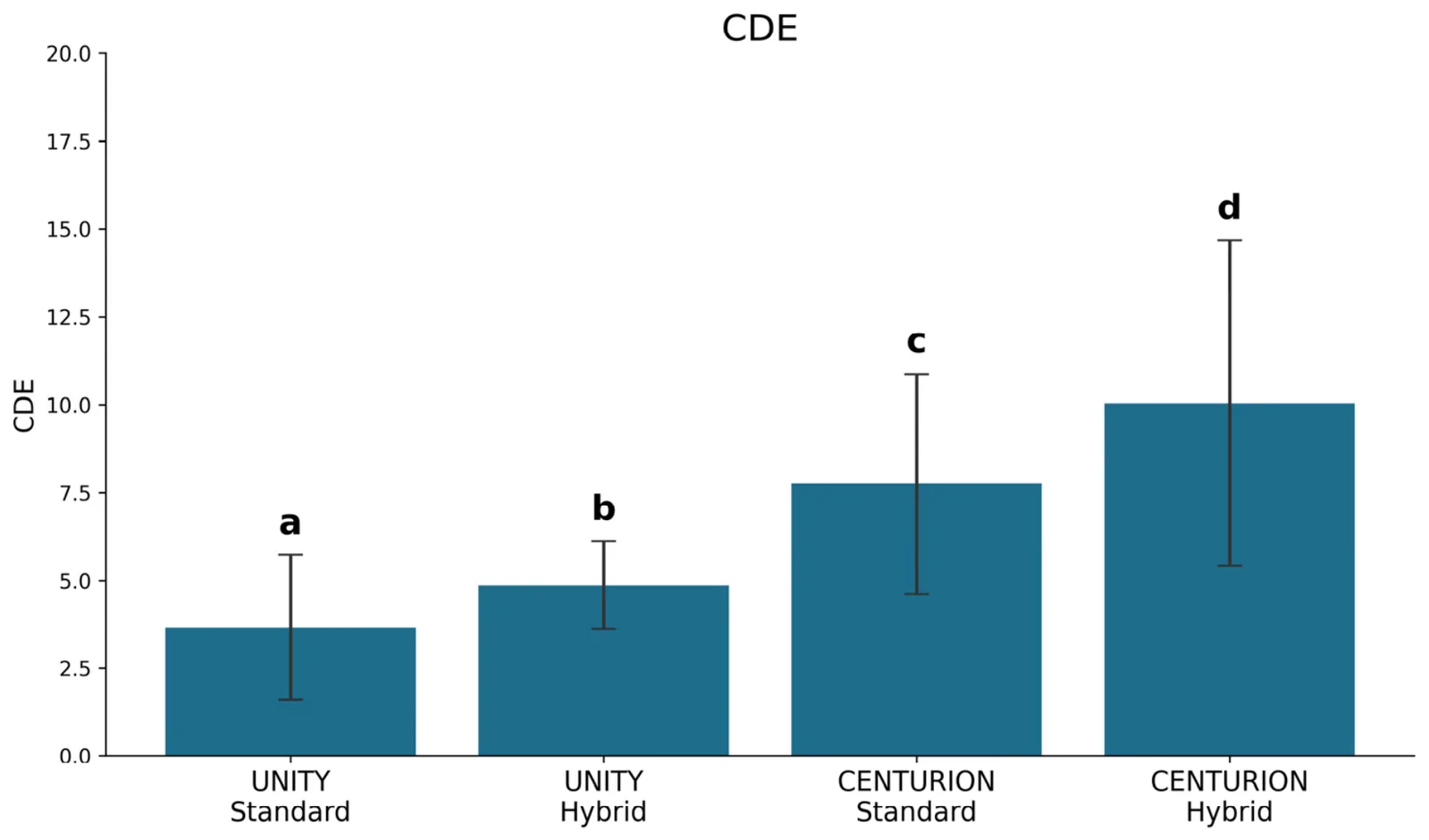
Cumulative dissipated energy (CDE) by device/tip group. Bars indicate mean; error bars indicate ±1 SD. Omnibus Kruskal–Wallis *p* < 0.0001. Groups sharing the same letter (a, b, c, d) are not significantly different on Bonferroni-adjusted pairwise Wilcoxon tests; all six pairwise comparisons were statistically significant (see Table 3 for exact *p* values).

Cumulative dissipated energy (CDE) differed markedly among the four groups (*p* < 0.0001; Figure 2), increasing progressively from the UNITY Standard group (3.66 ± 2.05) to the UNITY Hybrid group (4.87 ± 1.25) to the CENTURION Standard group (7.74 ± 3.14) to the CENTURION Hybrid group (10.04 ± 4.64). All six pairwise comparisons were statistically significant, including CENTURION Standard versus CENTURION Hybrid (*p* = 0.0375).

**Figure 3:**
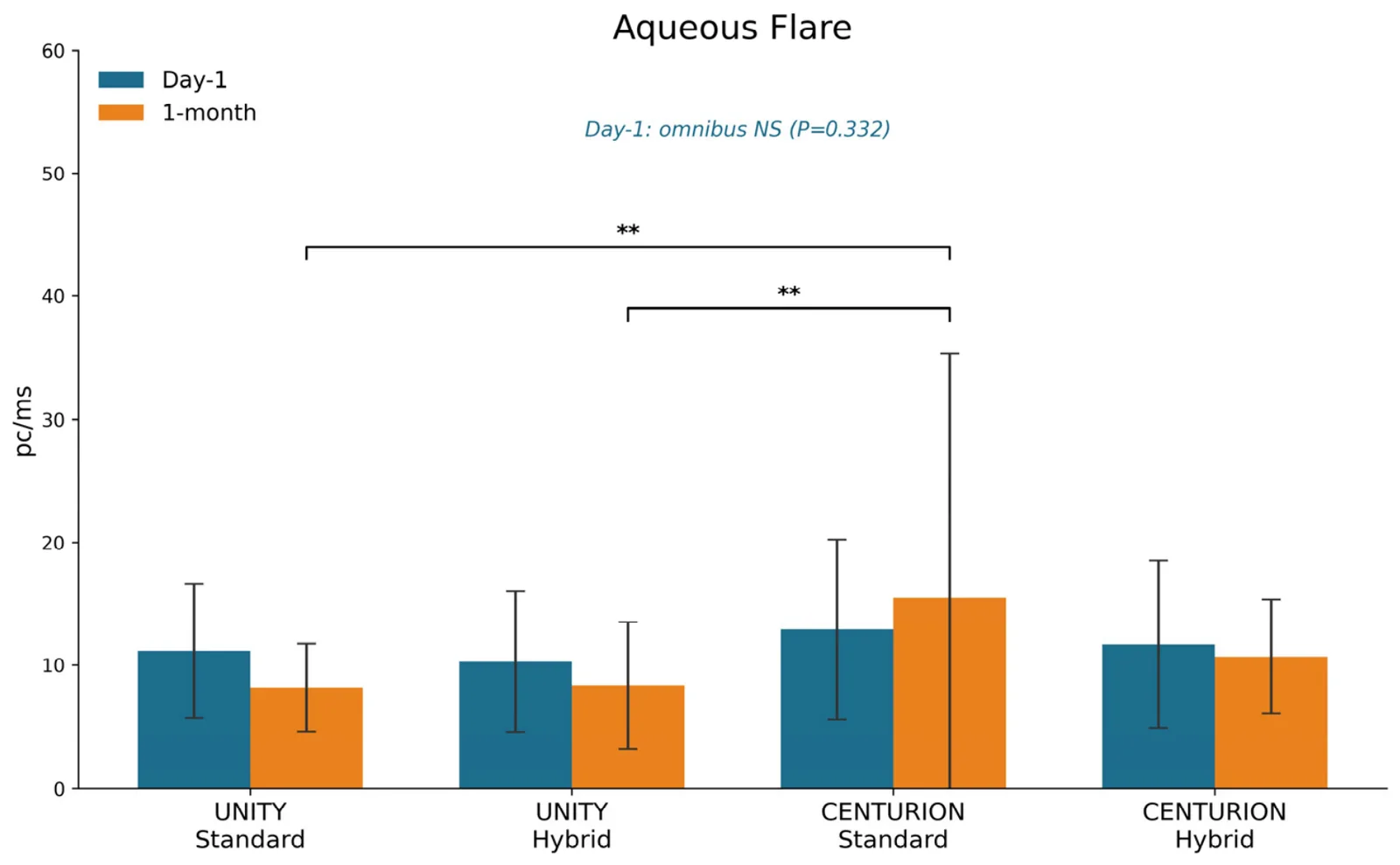
Postoperative aqueous flare (day-1 and 1-month flare), in photon counts per millisecond (pc/ms), by device/tip group. Bars indicate mean; error bars indicate ±1 SD. Day-1 flare did not differ significantly among groups (omnibus *p* = 0.332). For 1-month flare (omnibus *p* < 0.001), horizontal brackets denote statistically significant pairwise comparisons (Bonferroni-adjusted pairwise Wilcoxon tests; ** *p* < 0.01); all other 1-month pairs were not significant. See Table 3 for exact *p* values.

Postoperative 1-month aqueous flare differed significantly among the four groups (*p* < 0.001; Figure 3). Pairwise post hoc comparisons showed that the CENTURION Standard group had significantly higher 1-month flare values than both the UNITY Standard group (*p* = 0.0013) and the UNITY Hybrid group (*p* = 0.0034); no other pairwise comparison reached statistical significance.

**Figure 4:**
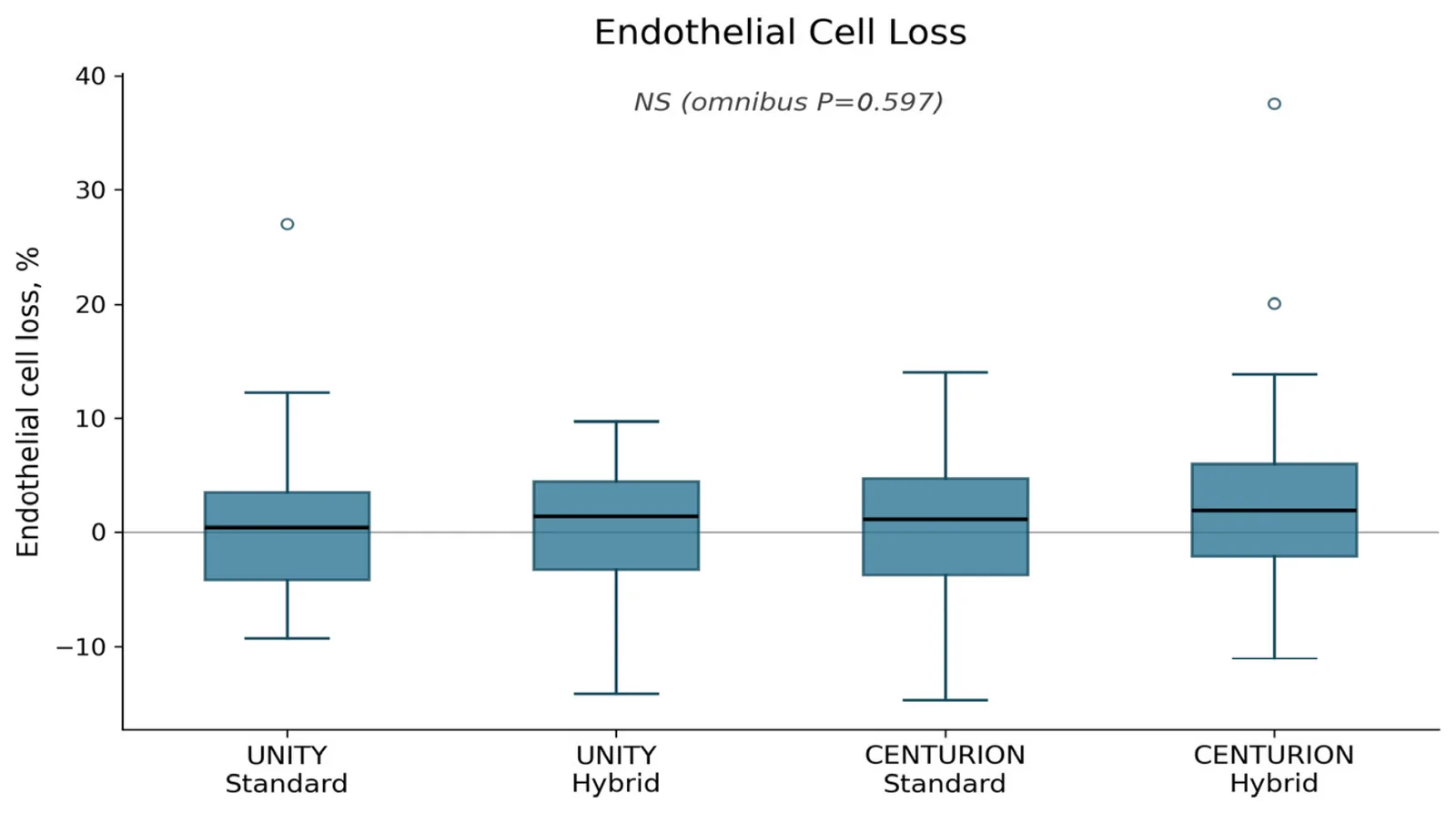
Endothelial cell loss (%) from baseline to 1 month by device/tip group. Boxes show the median and interquartile range (IQR); whiskers extend to the most extreme value within 1.5 × IQR; open circles denote outliers beyond this range. The omnibus difference among groups was not statistically significant (*p* = 0.597; NS).

At 1 month postoperatively, ECD did not differ significantly among the four groups (*p* = 0.350), nor did the percentage ECD decrease from baseline (*p* = 0.597), which averaged 0.9% ± 7.2% in the UNITY Standard group, 0.5% ± 5.4% in the UNITY Hybrid group, 0.7% ± 6.5% in the CENTURION Standard group, and 3.6% ± 9.4% in the CENTURION Hybrid group (Figure 4). Postoperative day-1 aqueous flare (*p* = 0.332) and 1-month CDVA (*p* = 0.326) likewise showed no significant between-group differences (Table 2).

Total surgical time also differed significantly on omnibus testing (*p* = 0.044); however, no individual pairwise comparison remained statistically significant after Bonferroni correction (all *p* ≥ 0.054), suggesting a weak and non-robust overall effect.

**Table 2:** Postoperative outcomes by device/technique group.

Outcome	UNITY Standard (*n* = 34)	UNITY Hybrid (*n* = 41)	CENTURION Standard (*n* = 47)	CENTURION Hybrid (*n* = 27)	*p* Value
1-month ECD, cells/mm^2^ (mean ± SD)	2701.8 ± 340.7	2814.0 ± 259.3	2738.7 ± 306.8	2647.7 ± 374.8	0.350
ECD decrease rate, % (mean ± SD)	0.9 ± 7.2	0.5 ± 5.4	0.7 ± 6.5	3.6 ± 9.4	0.597
Postoperative day-1 flare, pc/ms (mean ± SD)	11.17 ± 5.45	10.28 ± 5.71	12.90 ± 7.32	11.70 ± 6.80	0.332
Postoperative 1-month flare, pc/ms (mean ± SD; median [IQR])	8.18 ± 3.58 7.17 [5.76–8.89]	8.36 ± 5.15 7.61 [5.23–10.63]	15.45 ± 19.87 10.04 [8.02–15.70]	10.68 ± 4.64 9.50 [8.25–13.27]	<0.001
1-month CDVA, logMAR (mean ± SD)	−0.06 ± 0.03	−0.07 ± 0.03	−0.05 ± 0.09	−0.07 ± 0.03	0.326
CDE (mean ± SD)	3.66 ± 2.05	4.87 ± 1.25	7.74 ± 3.14	10.04 ± 4.64	<0.0001
Total surgical time, seconds (mean ± SD)	440.4 ± 153.7	437.6 ± 48.1	441.1 ± 85.4	463.4 ± 70.8	0.044

ECD = endothelial cell density; CDVA = corrected distance visual acuity; CDE = cumulative dissipated energy. All variables were compared with the Kruskal–Wallis test.

**Table 3:** Post hoc pairwise comparisons (Bonferroni-adjusted *p* values) for variables with significant omnibus tests.

Pairwise Comparison	1-Month Flare, *p*	CDE, *p*	Total Surgical Time, *p*
UNITY Standard vs. UNITY Hybrid	1.000	0.0003	0.219
UNITY Standard vs. CENTURION Standard	0.0013	<0.0001	1.000
UNITY Standard vs. CENTURION Hybrid	0.0798	<0.0001	0.054
UNITY Hybrid vs. CENTURION Standard	0.0034	<0.0001	1.000
UNITY Hybrid vs. CENTURION Hybrid	0.0812	<0.0001	0.805
CENTURION Standard vs. CENTURION Hybrid	1.000	0.0375	0.688

CDE = cumulative dissipated energy. For total surgical time, the omnibus Kruskal–Wallis test was significant (*p* = 0.044), but no pairwise comparison survived Bonferroni correction.

**Figure 5:**
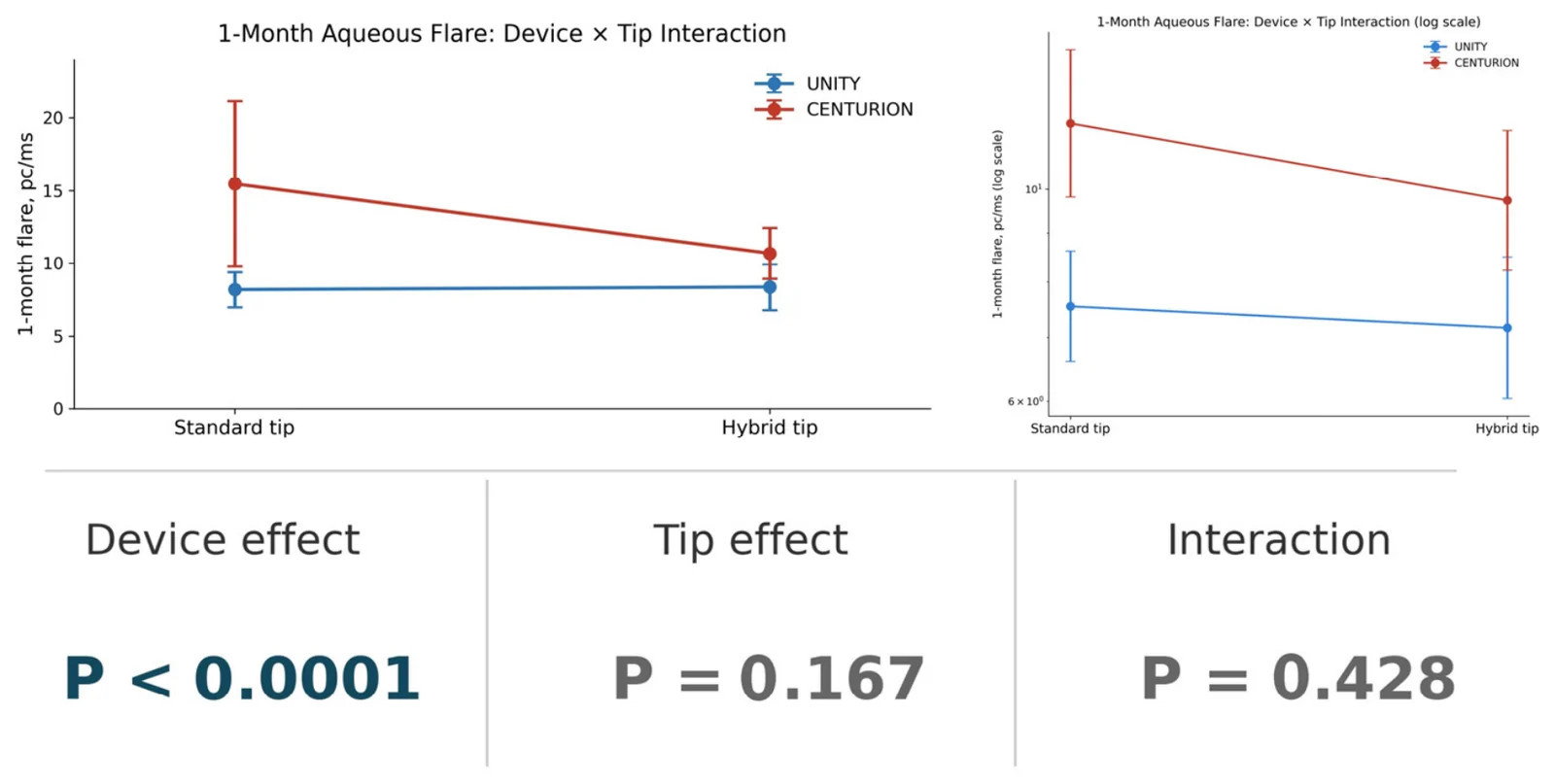
A 2 × 2 factorial analysis of 1-month aqueous flare (pc/ms) by device (UNITY vs. CENTURION) and tip (standard vs. hybrid). The left panel shows the original-scale mean and 95% CI for each device × tip combination; the right panel shows the same data as geometric means and log-scale 95% CI, matching the log-transformed model used for inference. The device effect was significant (*p* < 0.0001), the tip effect was not significant (*p* = 0.167), and the device × tip interaction was not significant (*p* = 0.428); the device × tip interaction being non-significant does not itself establish that the device effect is uniform across tip types.

The 2 × 2 factorial analysis (device × tip; Table 4) allowed the contribution of device and tip to be estimated separately from, and compared with, the four-group comparison above. For CDE, statistically significant associations were observed for both device and tip: UNITY was associated with a 52.8% lower geometric mean CDE than CENTURION (GMR 0.472; 95% CI, 0.413–0.539; *p* < 0.0001), while the hybrid tip was associated with a 35.9% higher geometric mean CDE than the standard tip (GMR 1.359; 95% CI, 1.190–1.551; *p* < 0.0001). The device × tip interaction was not statistically significant (*p* = 0.355). For 1-month aqueous flare, only device reached significance: UNITY was associated with a 31.6% lower geometric mean flare than CENTURION (GMR 0.684; 95% CI, 0.580–0.806; *p* < 0.0001; Figure 5), whereas the tip effect was not significant (*p* = 0.167) and neither was the interaction (*p* = 0.428). This divergence—device and tip both influencing CDE, but only device influencing flare—was consistent between the factorial model and the four-group comparisons in Table 2 and Table 3.

For the remaining outcomes, neither device nor tip reached statistical significance, and no device × tip interaction was detected (Table 4): 1-month ECD (device *p* = 0.328; tip *p* = 0.794; interaction *p* = 0.073), ECD decrease rate (device *p* = 0.316; tip *p* = 0.335; interaction *p* = 0.211), day-1 flare (device *p* = 0.243; tip *p* = 0.263; interaction *p* = 0.736), and total surgical time (device *p* = 0.264; tip *p* = 0.207; interaction *p* = 0.609). The tip effect on 1-month CDVA was AMD −0.015 logMAR (95% CI, −0.030 to 0.001; *p* = 0.071), while the device effect on CDVA (*p* = 0.387) and the device × tip interaction (*p* = 0.492) were not statistically significant.

**Table 4:** A 2 × 2 factorial analysis (device × tip) with HC3 robust standard errors.

Outcome	Scale	Device Effect (UNITY vs. CENTURION)	Tip Effect (Hybrid vs. Standard)	Interaction *p*
1-month ECD	Original	AMD 55.0 (−55.8 to 165.9), *p* = 0.328	AMD 14.7 (−96.2 to 125.7), *p* = 0.794	0.073
ECD decrease rate, %	Original	AMD −1.26 (−3.73 to 1.20), *p* = 0.316	AMD +1.22 (−1.26 to 3.71), *p* = 0.335	0.211
Day-1 flare, pc/ms	Log	GMR 0.905 (0.766–1.070), *p* = 0.243	GMR 0.909 (0.769–1.075), *p* = 0.263	0.736
1-month flare, pc/ms	Log	GMR 0.684 (0.580–0.806), *p* < 0.0001	GMR 0.891 (0.756–1.050), *p* = 0.167	0.428
1-month CDVA, logMAR	Original	AMD −0.008 (−0.025 to 0.010), *p* = 0.387	AMD −0.015 (−0.030 to 0.001), *p* = 0.071	0.492
CDE	Log	GMR 0.472 (0.413–0.539), *p* < 0.0001	GMR 1.359 (1.190–1.551), *p* < 0.0001	0.355
Total surgical time	Log	GMR 0.965 (0.908–1.027), *p* = 0.264	GMR 1.039 (0.979–1.104), *p* = 0.207	0.609

AMD = adjusted mean difference (original-scale outcomes); GMR = geometric mean ratio (log-transformed outcomes). Device reference = CENTURION; tip reference = standard. When the device × tip interaction was not significant, estimates are from the additive (main-effects) model.

To evaluate the impact of within-patient clustering (20 eyes from 10 patients, concentrated in the UNITY Standard and UNITY Hybrid groups), the seven factorial models in Table 4 were refitted with cluster-robust standard errors (patient as clustering unit) and, separately, after retaining only one randomly selected eye per patient (*n* = 139 eyes). Neither sensitivity analysis altered the significance status of any device or tip effect, and point estimates were materially unchanged from the primary analysis (e.g., 1-month ECD device effect: AMD 55.0 [95% CI, −56.3 to 166.4], *p* = 0.330 with cluster-robust standard errors, versus AMD 55.0 [95% CI, −55.8 to 165.9], *p* = 0.328 in the primary analysis; CDE device effect: GMR 0.472 [95% CI, 0.413–0.540], *p* < 0.0001 in both the cluster-robust and one-eye-per-patient analyses). Full sensitivity-analysis results are available from the authors upon request.

As a further sensitivity analysis, we fitted an ANCOVA model for 1-month ECD adjusted for baseline (preoperative) corneal endothelial density, with device, tip, and their interaction as predictors and HC3 robust standard errors. The device × tip interaction was not significant (*p* = 0.151), and in the additive model, baseline ECD was a strong independent predictor of 1-month ECD (β = 0.888 per cell/mm^2^, *p* < 0.0001), while neither the device effect (AMD 35.1 cells/mm^2^; 95% CI, −28.4 to 98.6; *p* = 0.276) nor the tip effect (AMD −26.6 cells/mm^2^; 95% CI, −92.7 to 39.5; *p* = 0.428) reached significance, consistent with the percentage-change analysis reported above.

## 4. Discussion

In this retrospective comparison of 149 eyes undergoing phacoemulsification with the UNITY 4D Phaco platform or the CENTURION torsional platform, each with a standard metal or hybrid polymer tip, UNITY was associated with substantially lower CDE and lower postoperative 1-month aqueous flare than CENTURION, while endothelial cell loss, day-1 flare, visual acuity, and surgical time did not differ significantly by device. The hybrid tip was associated with higher CDE than the standard tip, while no statistically significant device × tip interaction was detected for any outcome. Thus, the present data did not demonstrate statistically significant effect modification by tip type. Because the UNITY and CENTURION cohorts were drawn from non-overlapping calendar periods, these device-level findings should be read as a historical rather than a concurrent comparison; we return to this point, and its implications for interpretation, in the Limitations below.

The approximately 53% reduction in CDE with UNITY is in the same direction as bench comparisons of 4D Phaco and torsional ultrasound, which report roughly half the ultrasound time, energy, and total delivered energy for 4D Phaco relative to OZil torsional ultrasound, with improved microfollowability on bead-video analysis [1,3]. We have separately shown, using a bench model, that the UNITY platform maintains greater anterior chamber stability than CENTURION with Active Sentry across a range of simulated occlusion-break conditions [4]; this fluidic difference could plausibly extend to a fluidic advantage contributing to more consistent fragment engagement and lower repulsion-related energy loss during nucleus removal, although this link remains speculative and unconfirmed in the present dataset. A separate body of work has examined fluid dynamics and tip-to-tissue interaction comparing torsional and longitudinal ultrasound, rather than the 4D-versus-torsional comparison made here; that literature shows that non-longitudinal tip motion reduces fragment repulsion and chatter, with torsional ultrasound requiring substantially less surgeon-generated tip travel than longitudinal ultrasound [2], and high-speed videographic and particle-image-velocimetry analyses showing that lateral tip motion favorably alters cavitation and fluid streaming relative to longitudinal motion [16,17,18], consistent with bench comparisons of chatter and efficiency across non-longitudinal modalities [19,20]. While these torsional-versus-longitudinal mechanisms are not directly interchangeable with the 4D-versus-torsional comparison made here, they offer a plausible, though unproven, mechanistic framework. No statistically significant device × tip interaction was detected; however, this does not establish that the magnitude of the device association is uniform across tip types. The observed CDE difference may instead reflect differences between the UNITY and CENTURION systems as complete operating configurations, of which the 4D stroke is one element alongside platform-specific vacuum and aspiration flow settings that also differed between the two systems in this cohort, rather than the 4D stroke mechanism in isolation. This mechanistic literature is drawn largely from bench and ex vivo models, however, and our retrospective clinical dataset cannot itself establish causality for any single mechanism.

UNITY was also associated with an estimated 31.6% lower postoperative 1-month aqueous flare, a comparison that, to our knowledge, has not previously been made directly between 4D Phaco and torsional ultrasound platforms in a clinical cohort. Laser flare photometry provides an objective, quantitative measure of blood–aqueous barrier disruption and postoperative inflammatory stress, distinct from endothelial cell counts [12]. Preoperative flare and cataract density, rather than phacoemulsification energy itself, have been reported as the strongest predictors of postoperative blood–aqueous barrier damage [13], and the inflammatory response typically peaks within hours after surgery before resolving over subsequent days [15], a time course that may help explain why day-1 flare did not differ between devices in our cohort while 1-month flare did. The lower 1-month flare with UNITY may reflect lower cumulative ultrasound energy delivered to the eye, together with the greater anterior chamber stability we have previously described for this platform [4], more stable fragment engagement, and lower thermal load; the present design cannot isolate the relative contribution of each. Laser flare and cell photometry have also been used to stratify the risk of cystoid macular edema after cataract surgery [14], underscoring the potential clinical relevance of even modest differences in postoperative flare between platforms. Flare and ECD reflect biologically distinct aspects of surgical stress—inflammatory/vascular versus structural/mechanical—so these two outcomes need not move in parallel, as observed here.

Despite the marked reduction in CDE, UNITY was not associated with a statistically significant reduction in endothelial cell loss (adjusted difference, −1.3 percentage points; 95% CI, −3.7 to 1.2), suggesting that endothelial injury during phacoemulsification is multifactorial. Nuclear firmness has been identified as a stronger independent risk factor for endothelial cell loss than ultrasound energy itself, implicating direct mechanical fragment–endothelium contact [9]; shorter AL and longer phacoemulsification time are similarly implicated [11]. Comparative data across phaco-fragmentation techniques likewise show that lower CDE does not necessarily translate into lower endothelial cell loss [10], consistent with our findings; ACD and working space, irrigation turbulence, surgical manipulation, and the protective effect of OVDs likely also contribute. This nonsignificant result should not be interpreted as evidence of endothelial equivalence between platforms, because the study was not designed as an equivalence or noninferiority analysis; a substantially larger sample would be required to confidently exclude a small endothelial benefit or harm.

The hybrid polymer-coated tip was associated with higher CDE than the standard metal tip in our factorial model, consistent with a trade-off, reported on the bench, between the polymer coating’s capsular-contact safety profile and its ultrasound transmission efficiency: the hybrid tip tolerates significantly higher force before capsule rupture than a metal tip [5,6], and limited clinical experience has reported acceptable ultrasound parameters [7] and lower CDE with no capsule complications when combined with a pressure-sensing handpiece [8], albeit under different fluidic settings than used here. Higher CDE with the hybrid tip in our cohort should not be interpreted as overall inferiority: capsular safety, followability, and thermal behavior were not directly assessed here. The hybrid tip’s previously reported capsular safety advantage and its association with higher energy delivery in our cohort should therefore be regarded as separate, not necessarily conflicting, findings. The tip effect on 1-month CDVA was AMD −0.015 logMAR (95% CI, −0.030 to 0.001; *p* = 0.071). We did not directly measure followability or fragment engagement with the hybrid tip and therefore do not conclude that it alters these parameters. IOL selection (monofocal, enhanced monofocal, extended depth-of-focus, or trifocal, per surgeon and patient preference; Section 2.3) was not standardized or prospectively tracked by study group, and a reliable per-group optic-type breakdown could not be reconstructed retrospectively; unmeasured confounding by IOL type is therefore a limitation specifically for interpretation of the CDVA comparison, though it is less likely to affect CDE, flare, ECD, or surgical-time outcomes, which primarily reflect nucleus removal rather than lens implantation.

This study has several limitations. Most importantly, the CENTURION and UNITY platforms were not used concurrently: the CENTURION cohort was operated between September 2024 and January 2025, and the UNITY cohort between November 2025 and January 2026, separated by approximately 9 months during which UNITY’s fluidic settings, including intraocular pressure calibration, were being finalized for routine use (Section 2.1). The device comparison in this study is therefore better characterized as a historical (before-after) comparison than a concurrent one, and secular changes over this interval—including the surgeon’s accumulating experience, incremental adjustments to perioperative protocols, changes in the referred patient population, or other unmeasured practice-pattern drift—could confound the CDE, flare, or other differences attributed here to the device itself; the CDE and flare differences between UNITY and CENTURION should accordingly be regarded as hypothesis-generating pending confirmation in a design with concurrent or randomized device allocation. This concern applies specifically to the device comparison. In addition, because CDE is a console-reported value calculated by platform-specific algorithms (Section 2.4), our comparison assumes but does not independently establish that the two platforms’ CDE outputs are numerically equivalent measures of delivered ultrasound energy. Total ultrasound time was not separately recorded in this retrospective dataset and so could not be reported as an independent check on this assumption; only total surgical time (which includes non-ultrasound steps) is available (Table 2 and Table 4). CDE should accordingly be interpreted as a platform-reported metric rather than a directly validated measure of equivalent physical energy delivered. Standard-tip and hybrid-tip cases, by contrast, were performed over overlapping calendar periods within each device (CENTURION: hybrid-tip cases spanned September 2024–January 2025, encompassing the standard-tip period of December 2024–January 2025; UNITY: hybrid-tip cases began 15 December 2025, overlapping the final days of the standard-tip period before continuing through January 2026). For CENTURION, this substantial overlap limits temporal confounding of the tip comparison; for UNITY, however, the near-sequential enrollment of standard- and hybrid-tip cases (Section 2.1) means temporal confounding cannot be excluded for that platform’s tip comparison specifically, even though the tip comparison overall is less susceptible to this confound than the device comparison. Group allocation was not randomized overall, and group sizes were unequal; measured baseline characteristics are summarized in Table 1, but residual baseline imbalance and unmeasured confounding cannot be excluded. Follow-up was limited to 1 month; eligibility was restricted to Emery-Little grade 3 or lower to limit variability in lens density, but the exact grade distribution within this eligible range was not tabulated or compared across groups, and unmeasured confounding by surgical technique cannot be excluded. Twenty of the 149 eyes (from 10 patients) were paired within patients, concentrated in the UNITY Standard and UNITY Hybrid groups (Section 2.2). Cluster-robust and one-eye-per-patient sensitivity analyses (Section 3) yielded materially unchanged point estimates and did not alter the significance status of any outcome, suggesting this clustering did not substantially bias the primary results. Mechanistic evidence for the 4D stroke and for the hybrid tip is drawn largely from bench and ex vivo models, including our own anterior chamber stability data [4], and should not be assumed to translate directly into the clinical setting without further study. Finally, the factorial analysis, while informative for separating device and tip effects, had limited power to detect small interaction effects; the absence of a significant interaction should be interpreted as an absence of detected effect modification rather than proof that device and tip act entirely independently. Additionally, eyes with major intraoperative complications, including posterior capsule rupture and vitreous loss, were excluded by design. Consequently, the present study cannot assess whether platform or tip type influences complication risk, and the possibility of post-exposure selection bias cannot be excluded. Therefore, the findings should be interpreted as applying only to eyes that completed surgery without major intraoperative complications.

Taken together, reduced ultrasound energy did not necessarily translate into less endothelial injury in this cohort, but it was accompanied by a measurably lower postoperative inflammatory response. We did not formally compare the measurement sensitivity of flare photometry and endothelial cell counting, so we do not conclude that one is a more sensitive marker than the other; rather, these two outcomes showed different temporal and device-related patterns in this cohort, which may warrant further investigation into the determinants of postoperative aqueous flare independent of ultrasound energy.

## 5. Conclusions

In this retrospective, four-group and 2 × 2 factorial comparison, the UNITY 4D Phaco platform was associated with substantially lower cumulative dissipated energy and lower 1-month aqueous flare than the CENTURION torsional platform, without a demonstrable difference in endothelial cell loss or visual outcome at 1 month, and without significant modification by tip type. The hybrid polymer-coated tip was associated with higher CDE than the standard metal tip, with no statistically significant device × tip interaction detected, a finding that should be weighed against its previously reported capsular safety advantages rather than interpreted as overall inferiority. Because the device comparison reflects a historical rather than concurrent design (see Limitations), these findings—particularly the reduction in postoperative inflammatory flare—should be regarded as hypothesis-generating; prospective studies with concurrent or randomized device allocation, longer follow-up, and adequate power to detect device × tip interactions are warranted to confirm them and to clarify the underlying mechanisms.

## Data Availability

The data presented in this study are available on request from the corresponding author, owing to patient privacy restrictions.
